# Plus ça change? Switching lithium preparations

**DOI:** 10.1192/bjb.2021.126

**Published:** 2023-04

**Authors:** David A. Cousins, Thomas R. E. Barnes, Allan H. Young, Oriana Delgado, Carol Paton

**Affiliations:** 1 Newcastle University, Newcastle upon Tyne, UK; 2 Cumbria, Northumberland, Tyne and Wear NHS Foundation Trust, Newcastle upon Tyne, UK; 3 Imperial College London, UK; 4 Royal College of Psychiatrists, London, UK; 5 King's College London, UK; 6 South London & Maudsley NHS Foundation Trust, Beckenham, UK

**Keywords:** Priadel, Camcolit, plasma lithium levels, switch, adherence

## Abstract

**Aims and method:**

A supply disruption alert in 2020, now rescinded, notified UK prescribers of the planned discontinuation of Priadel^®^ (lithium carbonate) tablets. This service evaluation explored lithium dose and plasma levels before and after the switching of lithium brands, in order to determine the interchangeability of different brands of lithium from a pharmacokinetic perspective.

**Results:**

Data on the treatment of 37 patients switched from Priadel^®^ tablets were analysed. Switching to Camcolit^®^ controlled-release tablets at the same dose did not result in meaningful differences in plasma lithium levels. Dose adjustment and known or suspected poor medication adherence were associated with greater variability in plasma lithium levels on switching brands.

**Clinical implications:**

For comparable pre- and post-switch doses in adherent patients, the most common brands of lithium carbonate appear to produce similar plasma lithium levels. British National Formulary guidance relating to switching lithium brands may be unnecessarily complex.

The National Institute for Health and Care Excellence (NICE)^1,2^ recommends lithium for the treatment of mania and bipolar depression, the prevention of relapse in bipolar disorder and the augmentation of antidepressant medication in major depressive disorder. Lithium reduces suicidal behaviour^3^ and is licensed for the treatment of aggressive and self-mutilating behaviour.^4,5^ Lithium has a narrow therapeutic range^6,7^ and after titration and stabilisation of dose, plasma levels should be monitored at least every 6 months to ensure safe and effective prescribing.^1^ For most clinical indications, lithium is therapeutic at plasma levels somewhere between 0.4 and 1.0 mmol/L, with a narrower range commonly targeted for the optimal treatment of individual patients. Higher levels result in toxicity, which is not only concerning acutely but also associated with adverse long-term renal outcomes.^8^ Levels below 0.4 mmol/L are unlikely to be effective and increase the risk of relapse.^9^

In the UK, Priadel^®^ (lithium carbonate) modified-release tablets account for 90% of lithium prescriptions.^10^ In 2020, the failure to agree a National Health Service (NHS) pricing structure for Priadel^®^ tablets led to the manufacturers serving notice of their intention to cease production. A supply disruption alert (SDA) was therefore issued by the Department of Health and Social Care and NHS England in August 2020 (SDA/2020/012),^11^ outlining the need to switch patients from Priadel^®^ tablets to alternative formulations of lithium. Of the alternative solid dosage formulations available in the UK, and as described by the manufacturers, Camcolit^®^ 400 mg and Liskonum^®^ 450 mg are controlled-release preparations, whereas lithium carbonate Essential Pharma^®^ 250 mg is immediate release (Box 1). Given the paucity of comparative pharmacokinetic data for alternative preparations, the inability to halve some brands of tablets within licence and the British National Formulary (BNF) recommendations to apply the same precautions to switching as for initiation (twice-daily dose titration in some instances), the SDA offered pragmatic advice: measure the 12 h plasma lithium level before switching, switch to the nearest possible dose of another formulation ensuring no interruption to treatment while maintaining the previous dosing schedule, and measure the 12 h plasma lithium level 7 days after switching.

Box 1 Definitions of formulations.

*Immediate release*

Formulated to dissolve without delaying or prolonging dissolution or absorption

*Modified release*

Formulated to delay or extend or alter the site of release of medication

*Controlled release*

Formulated to release medication in a controlled manner over an extended period of time

We planned a survey to assess whether the pragmatic advice for switching lithium brands was being followed and the consequences of this. However, within 6 weeks of the original SDA being issued, it was rescinded and so there was no longer any urgent need for clinicians to switch patients from Priadel^®^ tablets. Nevertheless, appraising the switches that had been initiated soon after the SDA release provided an opportunity to add to the limited data available regarding the interchangeability of lithium brands, so we proceeded with data collection.

## Method

The Prescribing Observatory for Mental Heath (POMH) used its established infrastructure to invite mental health services to submit data relating to their early experiences of switching lithium formulations. It further clarified that the exercise was a stand-alone service evaluation rather than part of a POMH quality improvement programme. Ethical approval is not required for such initiatives.^12^

A bespoke data collection form was developed in collaboration with POMH to facilitate the systematic collection of the relevant information and this was distributed to all POMH member mental health services, along with an explanation of the rationale for the collection of these data. The patient information collected was age, gender, ethnicity and psychiatric diagnosis. Regarding medication, pre-switch information was gathered on the dose of Priadel^®^ tablets, the prescription regimen, the most recent plasma lithium level and any known or suspected issues with medication adherence. Post-switch, the data collected were the brand of lithium prescribed, the daily dose and regimen, and the plasma lithium level.

Each mental health service was invited to share the data collection tool with their chief pharmacist and to complete the form, using information from the clinical records, for each eligible patient, namely any patient who had been switched, as part of their routine clinical care, from Priadel^®^ tablets to alternative formulations of lithium because of its planned discontinuation. All patients who had undergone such a switch were eligible. The practicalities of patient identification and data collection were left to the discretion of the individual participating services. The data were submitted online using Formic software^13^ – the data submitted were pseudonymous within each mental health service and anonymous for the analysis within POMH.

The data collection period was originally planned for five weeks, from October to November 2020, but with the expectation that fewer cases were likely to be switched following the rescinding of the SDA, this was extended for a further month.

A descriptive statistical analysis was conducted using SPSS v26.^14^ Results are expressed as means and standard deviations unless otherwise specified.

## Results

Fifteen POMH member trusts submitted data for 41 eligible patients. The demographic and diagnostic characteristics of this patient sample are presented in Table 1. The brands of lithium to which these patients had been switched are listed in Table 2. The data on 37 patients were considered suitable for further descriptive analysis and the results presented henceforth relate to this subgroup. The reasons for excluding four patients were as follows: two patients had partial missing data (post-switch plasma lithium level), one had been switched from Priadel^®^ liquid and one had been switched to lithium liquid. Liquid formulations contain lithium citrate and the differences in bioavailability and absorption rate characteristics complicate comparisons with lithium carbonate formulations.

**Table 1:** Demographic and diagnostic characteristics of the patient sample

Demographic and diagnostic variables	Total sample (*n* = 41)	Lithium level comparison subsample (*n* = 37)
Gender, *n* (%)		
Male	16 (39)	14 (39)
Female	25 (61)	23 (61)
Ethnicity, *n* (%)		
White/White British	38 (93)	34 (92)
Black/Black British	3 (7)	3 (8)
Age band, years: *n* (%)		
19–25	2 (5)	1 (3)
26–35	10 (24)	9 (24)
36–45	5 (12)	5 (14)
46–55	6 (15)	6 (16)
56–65	8 (20)	7 (19)
>65	10 (24)	9 (24)
Median age, *n* (%)	53	53
Age range, years: *n* (%)	24–84	24–84
Psychiatric diagnosis, *n* (%)		
Bipolar disorder	22 (54)	19 (51)
Unipolar depression	9 (22)	8 (22)
Schizophrenia/schizoaffective	9 (22)	9 (24)
Other	1 (2)	1(3)

**Table 2:** Brands of lithium to which patients were switched

	*n* (%)	
Lithium brand following switch	Total sample (*n* = 41)	Lithium level comparison subsample (*n* = 37)
Single		
Camcolit^®^ 400 mg	30 (73)	27 (73)
Essential Pharma^®^ 250 mg	5 (12)	5 (14)
Liskonum^®^ 450 mg	2 (5)	2 (5)
Lithium citrate liquid	1 (2)	—
Combination		
Camcolit^®^ 400 mg and Essential Pharma^®^ 250 mg	3 (7)	3 (8)

### Priadel^®^ tablet dosage and plasma levels prior to switching

The median daily dose of Priadel^®^ tablets was 600 mg (range 200–1300 mg). The mean plasma lithium level prior to switching was 0.60 mmol/L (s.d. = 0.22; range 0.10–1.10 mmol/L), and five patients had levels outside the accepted therapeutic range of 0.4–1.0 mmol/L. Nine patients (24%) had known or suspected issues with adherence to their medication regimens.

### Lithium brands and dosage to which patients were switched

Of the 37 patients, 27 (73%) had been switched from Priadel^®^ to Camcolit^®^ 400 mg tablets and a directly comparable dose in milligrams was achieved in 23 cases. Five patients had been switched to lithium carbonate Essential Pharma^®^ 250 mg tablets, with four undergoing a dose change and one remaining on the same dose. Two patients had been switched to Liskonum^®^ tablets, one from 600 mg Priadel^®^ to 675 mg Liskonium^®^ and the other from 400 mg Priadel^®^ to 900 mg of Liskonium^®^ tablets (in divided doses). Three patients had been switched to a combination of Camcolit^®^ 400 mg and lithium carbonate Essential Pharma^®^ 250 mg tablets, with two undergoing a small change in dose.

Of the nine patients who had been switched from a Priadel^®^ dose of 600 mg, three were prescribed 600 mg of Camcolit^®^, which presumably involved the halving of 400 mg tablets. In three other patients, the dose was reduced to 500 mg (using lithium carbonate Essential Pharma^®^ 250 mg tablets). In the other three, the dose was increased: in one patient to 675 mg (using Liskonum^®^ tablets), in another to 800 mg (using Camcolit^®^ 400 mg tablets) and in a third patient to 650 mg by combining brands (Camcolit^®^ 400 mg and lithium carbonate Essential Pharma^®^ 250 mg tablets).

Eleven patients (30%) had been switched from a dose of Priadel^®^ tablets that could not be achieved using either an alternative single brand within licence or a combination of brands (600 mg, *n* = 9; 300 mg, *n* = 1; 200 mg, *n* = 1). For 8 of these 11 patients, the dose was changed on switching; 2 of these 8 patients had pre-switch plasma lithium levels outside the 0.4–1.0 mmol/L range.

### Plasma lithium levels after switching

**Fig. 1:**
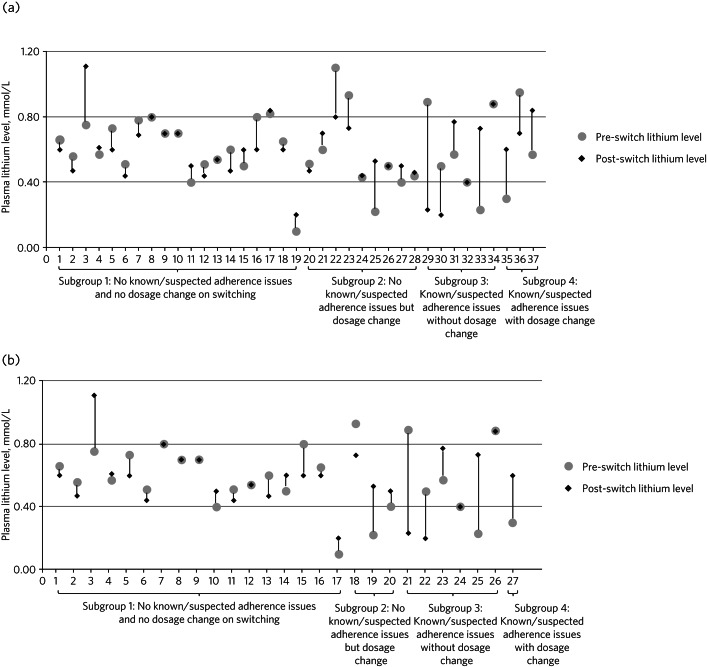
Individual pre- and post-switch plasma lithium levels by *post hoc* group allocation according to dose change and adherence issue status for (a) all patients (*n* = 37) and (b) patients switched to Camcolit^®^ tablets (*n* = 27).

The post-switch plasma lithium level had been checked within 1 week of switching in 13 (35%) patients, and within 3 weeks in 33 (95%) patients. The pre- and post-switch plasma lithium levels for the 37 patients are shown in Fig. 1(a), in four subgroups. Subgroup 1 (*n* = 19) included patients for whom there were no apparent adherence issues and no dosage change on switching. The patients in subgroup 2 (*n* = 9) also had no adherence issues but there had been a change in dosage on switching. Subgroup 3 (*n* = 6) included those patients with a known or suspected adherence issue but no change in dose on switching and the patients in subgroup 4 (*n* = 3) had known or suspected adherence issues and a change in dose.

Those patients with known or suspected adherence issues and/or a dosage change (subgroups 2, 3 and 4) appeared to have greater variation in plasma lithium levels both before and after switching, compared with the patients in subgroup 1, who had neither adherence issues nor dosage change. A similar pattern was observed in the 27 patients who were switched from Priadel^®^ to Camcolit^®^ 400 mg tablets, proportionately fewer of whom underwent a dose change (Fig. 1(b)).

## Discussion

In this case series, the majority of switches from Priadel^®^ tablets were to Camcolit^®^ 400 mg tablets and this rarely led to unexpectedly high or low post-switch plasma lithium levels. However, known or suspected issues with medication adherence were present in a quarter of cases and as might be expected, pre- and post-switch plasma lithium levels appeared to vary more in these patients than in those with no known adherence issues.

Single daily dosing was maintained on switching from Priadel^®^ tablets to another brand of lithium for all but one patient. In around a third of patients, the switch involved a dosage change but, in these instances, only a minority had pre-switch plasma lithium levels outside the therapeutic range. The decision to change dosage may reflect that some clinicians preferred to use an alternative brand of lithium using whole tablets rather than breaking Camcolit^®^ 400 mg tablets in half to achieve an equivalent dose. This may be because halving the tablets would constitute unlicensed use and/or be an inconvenient or impractical task for some patients.

### What is known about the pharmacokinetics of different solid dosage formulations of lithium carbonate?

Besides Priadel^®^ tablets, there are three solid dosage formulations of lithium carbonate currently available in the UK: Camcolit^®^ 400 mg tablets and Liskonium^®^ 450 mg tablets, both of which are controlled-release preparations, and lithium carbonate Essential Pharma^®^ 250 mg tablets, which are immediate release. There are very few published studies that compare the pharmacokinetics of different formulations of lithium but those that do exist are very small and the varying methodologies used make it difficult to draw firm conclusions.^15–18^ Nevertheless, these studies suggest that, using the same dose, Priadel^®^, Camcolit^®^ and Liskonium^®^ give broadly similar 12 h plasma lithium levels, whereas Phasal^®^ (no longer available in the UK) does not.

### What does the BNF recommend when switching brands?

The BNF states that lithium preparations vary widely in bioavailability and that changing the preparation requires the same precautions as initiation of treatment.^4^ It also strongly discourages the generic prescribing of lithium and patients are advised that they should always check their prescription to ensure that they have received the correct brand.

When starting treatment with solid dosage formulations other than Priadel^®^ tablets, the BNF recommends that lithium should be administered in divided doses with the dose adjusted according to the 12 h plasma lithium level, followed by a switch to once-daily dosing when the plasma level is stable within the desired range.

### When and why were these recommendations made?

Lithium carbonate as Camcolit^®^ 250 mg tablets (immediate release) first appeared in the edition of the BNF that covered the years 1976 to 1978 and was the only lithium formulation listed. By the time the next edition was published in 1980, three controlled-release formulations (Camcolit^®^ 400 mg, Priadel^®^ and Phasal^®^) were also listed along with the now familiar statement ‘different preparations vary widely in bioavailability’. This statement was likely informed by the small pharmacokinetic studies already mentioned,^15,17^ in which Phasal^®^ was noted to give very different 12 h plasma levels to Camcolit^®^ and Priadel^®^ tablets, the findings from which were incorporated into the summary of product characteristics (SmPC). Phasal^®^ is no longer available in the UK, but the relevant statements in SmPCs and the warning statement in the BNF remain unchanged, perhaps because of the lack of new data – to our knowledge, there have been no studies comparing the pharmacokinetics of different solid dose lithium formulations in the last 30 years.

### Concerns raised by the planned discontinuation of Priadel^®^ tablets

The proposed discontinuation of Priadel^®^ tablets and consequent need to switch a large numbers of patients in a relatively short period of time exposed two main areas of uncertainty. First, whether Camcolit^®^ 400 mg tablets should be halved for the purposes of achieving the desired dose and, second, whether the long-standing recommendations in the BNF, discussed above, should be strictly followed.

### Breaking Camcolit^®^ 400 mg tablets in half

Priadel^®^ tablets (200 and 400 mg) are licensed to be halved for the purpose of dose adjustment. Liskonum^®^ likewise, but with a single formulation of 450 mg there are fewer dose increments available. Camcolit^®^ 400 mg tablets are not licensed to be halved for the purpose of administering half the dose, but the tablets have a score line to allow division for ease of swallowing. The manufacturers of Camcolit^®^ 400 mg tablets state that there is no supporting information available to demonstrate that by dividing the tablets along the score line, exactly half the dose is provided, but confirm that the active ingredient (lithium carbonate) is distributed evenly throughout the tablet core and the controlled-release mechanism is not affected by splitting the tablet (M Mason, personal communication, 2022). The same applies to lithium carbonate Essential Pharma^®^ 250 mg, but these smaller tablets are more difficult to divide evenly, and this brand of lithium has a different, immediate-release formulation.

The SDA anticipated that certain common doses of Priadel^®^ tablets, such as 600 mg daily, would present a challenge for comparable dose selection. Even in the small service evaluation presented here, five different switching strategies were observed in the nine patients taking this dose. Were this to be representative of more widespread practice, such complexity of switching could lead to numerous errors at the level of prescribers, dispensing pharmacies, and individual patients. Where patients do not find breaking tablets in half to be problematic, we suggest that the strategy of dividing Camcolit^®^ 400 mg tablets should be employed if switching from Priadel^®^ tablets is required in the future.

### Advice in the SDA relating to switching brands of lithium

As described above, the limited data available suggest that the controlled-release solid dose formulations of lithium carbonate that are currently available in the UK give very similar 12 h plasma lithium levels,^18^ so the SDA recommended a simple switch to the same dose, maintaining once daily dosing. It was noted that the pharmacokinetics of Camcolit^®^ 400 mg tablets were nearest to those of Priadel^®^ and although the SDA stopped short of recommending that all patients were switched to this preparation, three-quarters of our small sample were. The advice to maintain once daily dosing was followed in all but one case.

### Strengths and limitations

Although small, this service evaluation represents the largest case series to date, providing data on plasma lithium levels before and after the switching of solid dosage lithium carbonate brands. Data were collected as part of routine clinical care and the decisions to switch and submit data were made by clinicians. The sample might therefore not be representative of the wider patient population taking lithium. However, the demographic and diagnostic characteristics of our sample are consistent with those of large audit samples of patients prescribed lithium, collected in the context of quality improvement programmes conducted by POMH.^19^ In measuring plasma lithium levels, data that would have allowed the influence of variables such as the timing of blood samples, levels of hydration and changes in interacting medications to be explored were not collected. There was no objective measure of medication adherence pre- or post-switch.

### Research and clinical implications

There is a need for formal systematic research to evaluate the interchangeability of solid dosage lithium carbonate brands, controlling for factors such as adherence, the timing of blood samples, levels of hydration and the influence of interacting medications, and to explore the influence of demographic and clinical characteristics such as gender, diagnosis and renal function on lithium pharmacokinetics.

The results of this service evaluation demonstrate that the pragmatic advice on switching from Priadel^®^ to another lithium brand given in the SDA rarely led to unexpectedly high or low post-switch plasma lithium levels. Lithium levels remained within the recommended range in the majority of cases, including those where a dose approximation was required. Patients with known or suspected issues with adherence had significantly greater variability in plasma lithium levels on switching, and closer monitoring would be advisable in such cases if switching is required. Further, our findings prompt caution regarding anticipatory changes in dose when switching lithium formulations in patients with plasma lithium levels outside the recommended range and, rather, support swift review and adjustment of dose according to post-switch levels. If wholesale switching from Priadel^®^ tablets in the UK were to be required in the future, consistent guidance supporting the halving of Camcolit^®^ 400 mg tablets might reduce the need for unnecessary dose adjustments and brand mixing in some patients.

## Data Availability

The authors confirm that the data supporting the findings of this study are available within the article.

## About the authors

**David A. Cousins**, Translational and Clinical Research Institute, Newcastle University, Newcastle upon Tyne; and Regional Affective Disorders Service, Cumbria, Northumberland, Tyne and Wear NHS Foundation Trust, Newcastle upon Tyne, UK. **Thomas R. E. Barnes**, Division of Psychiatry, Imperial College London; and Prescribing Observatory for Mental Health, Royal College of Psychiatrists, London, UK. **Allan H. Young**, Department of Psychological Medicine, Institute of Psychiatry, Psychology and Neuroscience, King's College London; and South London & Maudsley NHS Foundation Trust, Bethlem Royal Hospital, Beckenham, UK. **Oriana Delgado**, Prescribing Observatory for Mental Health, Royal College of Psychiatrists, London, UK. **Carol Paton**, Division of Psychiatry, Imperial College London; and Prescribing Observatory for Mental Health, Royal College of Psychiatrists, London, UK.

## References

[1] National Institute for Health and Care Excellence. Bipolar Disorder: Assessment and Management (CG185). NICE, 2020. 31556980

[2] National Institute for Health and Care Excellence. Depression in Adults: Recognition and Management (CG90). NICE, 2009. 31990491

[3] Lewitzka U, Severus E, Bauer R, Ritter P, Muller-Oerlinghausen B, Bauer M. The suicide prevention effect of lithium: more than 20 years of evidence-a narrative review.Int J Bipolar Disord 2015; 3(1): 15. 10.1186/s40345-015-0032-2 PMC4504869 26183461

[4] Joint Formulary Committee. Lithium carbonate. In *British National Formulary (BNF)*. BMJ Publishing Group/Royal Pharmaceutical Society (https://bnf.nice.org.uk/drug/lithium-carbonate.html#indicationsAndDoses [cited Mar 2021]).

[5] Umukoro S, Aladeokin AC, Eduviere AT. Aggressive behavior: a comprehensive review of its neurochemical mechanisms and management. Agress Violent Beh 2013; 18(2): 195–203.

[6] Malhi GS, Tanious M, Gershon S. The lithiumeter: a measured approach. Bipolar Disord 2011; 13: 219–26. 21676125 10.1111/j.1399-5618.2011.00918.x

[7] Paton C, Barnes TRE, Shingleton-Smith A, McAllister-Williams RH, Kirkbride J, Jones PB, Lithium in bipolar and other affective disorders: prescribing practice in the UK. J Psychopharmacol 2010; 24: 1739–46. 20488832 10.1177/0269881110367728

[8] Clos S, Rauchhaus P, Severn A, Cochrane L, Donnan PT. Long-term effect of lithium maintenance therapy on estimated glomerular filtration rate in patients with affective disorders: a population-based cohort study. Lancet Psychiatry 2015; 2: 1075–83. 26453408 10.1016/S2215-0366(15)00316-8

[9] Severus WE, Kleindienst N, Seemüeller F, Frangou S, Möeller HJ, Greil W. What is the optimal serum lithium level in the long-term treatment of bipolar disorder - a review? Bipolar Disord 2008; 10 231–7. 18271901 10.1111/j.1399-5618.2007.00475.x

[10] Phizackerley D, Cave J. What price Priadel? Drug Ther Bull 2020; 58: 179–80. 33158854 10.1136/dtb.2020.000066

[11] Medicines & Healthcare products Regulatory Agency. Lithium Carbonate (Priadel) 200mg and 400mg Modified Release Tablets - Supply Disruption. MHRA, 2020 (https://www.cas.mhra.gov.uk/ViewandAcknowledgment/ViewAlert.aspx?AlertID=103087.

[12] Health Research Authority. Defining Research – Do I Need NHS Ethics Approval? HRA, 2017 (http://www.hra-decisiontools.org.uk/research/docs/DefiningResearchTable_Oct2017-1.pdf).

[13] FormicSolutions. FormicSolutions, 2016. (http://www.Formic.com/survey-software/).

[14] IBM. SPSS Statistics for Mac v26. IBM Corp, 2019.

[15] Bennie EH, Manzoor AKM, Scott AM, Fell GS. Serum concentrations of lithium after 3 proprietary preparations of lithium-carbonate (Priadel, Phasal and Camcolit). Br J Clin Pharmacol 1977; 4: 479–83. 901741 10.1111/j.1365-2125.1977.tb00766.x PMC1429041

[16] Johnson GFS, Hunt GE. Pharmacokinetics of lithium preparations in patients. Prog Neuropsychopharmacol Biol Psychiatry 1984; 8: 63–70. 6427848 10.1016/0278-5846(84)90136-2

[17] Tyrer S, Hullin RP, Birch NJ, Goodwin JC. Absorption of lithium following administration of slow-release and conventional preparations. Psychol Med 1976; 6: 51–8. 935297 10.1017/s0033291700007492

[18] Shelley RK, Silverstone T. Single dose pharmacokinetics of 5 formulations of lithium - a controlled comparison in healthy-subjects. Int Clin Psychopharmacol 1986; 1: 324–31. 3104448 10.1097/00004850-198610000-00007

[19] Paton C, Adroer R, Barnes TRE. Monitoring lithium therapy: the impact of a quality improvement programme in the UK. Bipolar Disord 2013; 15(8): 865–75. 24119180 10.1111/bdi.12128

